# Author Correction: A two-dimensional mid-infrared optoelectronic retina enabling simultaneous perception and encoding

**DOI:** 10.1038/s41467-023-43859-y

**Published:** 2023-11-30

**Authors:** Fakun Wang, Fangchen Hu, Mingjin Dai, Song Zhu, Fangyuan Sun, Ruihuan Duan, Chongwu Wang, Jiayue Han, Wenjie Deng, Wenduo Chen, Ming Ye, Song Han, Bo Qiang, Yuhao Jin, Yunda Chua, Nan Chi, Shaohua Yu, Donguk Nam, Sang Hoon Chae, Zheng Liu, Qi Jie Wang

**Affiliations:** 1 School of Electrical and Electronic Engineering, Nanyang Technological University, Singapore, 639798 Singapore; 2 Key Laboratory for Information Science of Electromagnetic Waves (MoE), Fudan University, Shanghai, 200433 China; 3 School of Materials Science and Engineering, Nanyang Technological University, Singapore, 639798 Singapore; 4 Peng Cheng Laboratory, Shenzhen, 518055 China; 5 Centre for Disruptive Photonic Technologies, School of Physical and Mathematical Sciences, Nanyang Technological University, Singapore, 637371 Singapore

**Keywords:** Materials for devices, Optical materials and structures

Correction to: *Nature Communications* 10.1038/s41467-023-37623-5, published online 06 April 2023

In the Acknowledgements section of this article, the grant number relating to the National Medical Research Council given for Qi Jie Wang was incorrectly given as 021528-00001 and should have been MOH-000927. The original article has been corrected.

