# Supplementary material for: A two-dimensional mid-infrared optoelectronic retina enabling simultaneous perception and encoding
Source: Nat Commun. 2023 Apr 6;14:1938. doi: 10.1038/s41467-023-37623-5 (PMC10079931; doi:10.1038/s41467-023-37623-5)
Supplement: Supplementary file 1 — Supplementary information [file 41467_2023_37623_MOESM1_ESM.pdf]

Supplementary Information

A Two-dimensional Mid-Infrared Optoelectronic Retina Enabling Simultaneous Perception and Encoding

Fakun Wang,[1,†] Fangchen Hu,[1,2,†] Mingjin Dai,[1] Song Zhu,[1] Fangyuan Sun,[1] Ruihuan Duan,[3] Chongwu Wang,[1] Jiayue Han,[1] Wenjie Deng,[1] Wenduo Chen,[1] Ming Ye,[1] Song Han,[1] Bo Qiang,[1] Yuhao Jin,[1] Yunda Chua,[1] Nan Chi,[2] Shaohua Yu,[4] Donguk Nam,[1] Sang Hoon Chae,[1] Zheng Liu,[3] Qi Jie Wang[1,5 *]

[1]School of Electrical & Electronic Engineering, Nanyang Technological University, Singapore 639798, Singapore.

[2]Key Laboratory for Information Science of Electromagnetic Waves (MoE), Fudan University, Shanghai 200433, China.

[3]School of Materials Science and Engineering, Nanyang Technological University, Singapore 639798, Singapore.

[4]Peng Cheng Laboratory, Shenzhen 518055, China

[5]Centre for Disruptive Photonic Technologies, School of Physical and Mathematical Sciences, Nanyang Technological University, Singapore 637371, Singapore

[†]These authors contributed equally: Fakun Wang, Fangchen Hu.

*Corresponding author. Email: qjwang@ntu.edu.sg

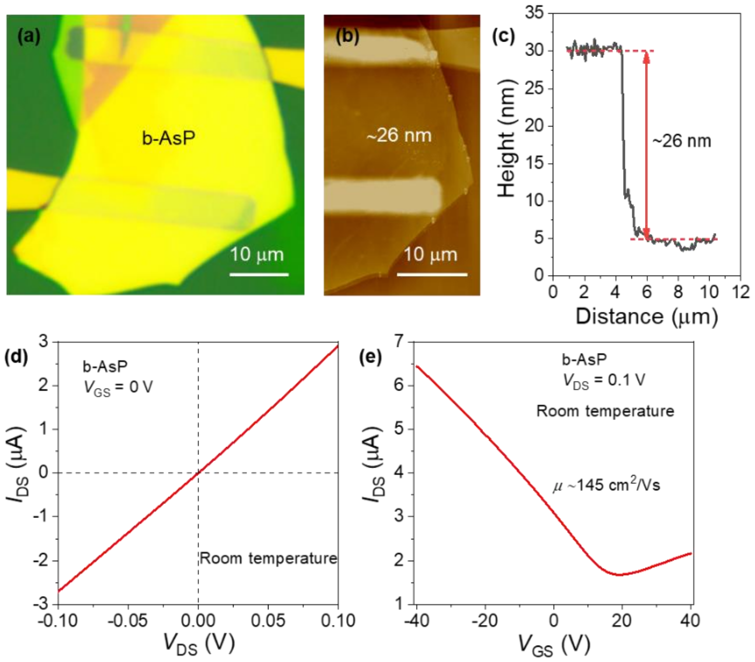

Supplementary Fig. 1 Morphology characterization and electrical properties of b-AsP. (a) Optical image and (b) atomic force microscopic image of a 2D b-AsP-based back-gate field-effect transistor. (c) Height profile of the 2D b-AsP flake, showing a thickness of ~26 nm. (d) Typical I_{DS}-V_{DS} curve of a 2D b AsP-based transistor. Linear I_{DS}-V_{DS} curve indicates that Ohmic contact was formed between the 2D b-AsP flake and Au electrodes. (e) I_{DS}-V_{GS} curve at V_{DS} = 0.1 V. It can be observed that the b-AsP exhibits heavily-doped p-type conduction behavior with a high hole mobility of 145 cm^2/Vs at room temperature.

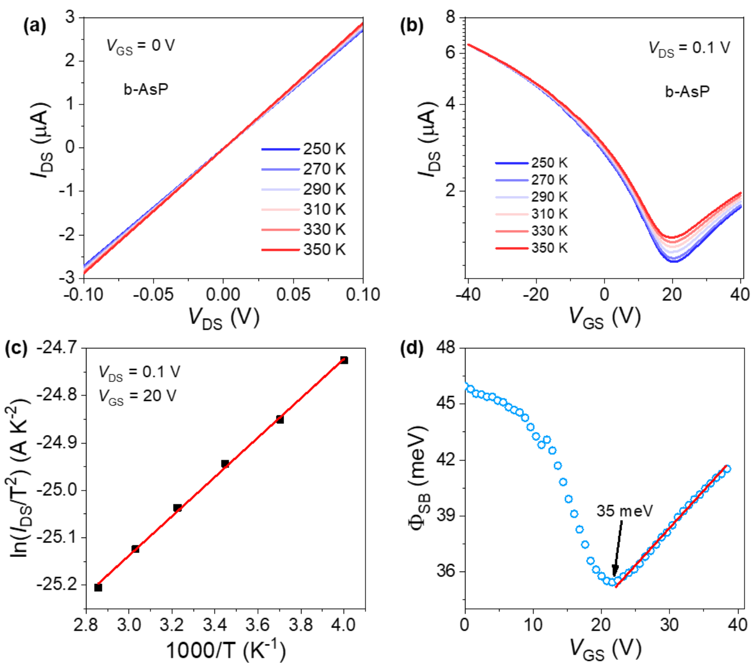

Supplementary Fig. 2 Temperature-dependent electrical characteristics of b-AsP transistor. (a) I_{DS}-V_{DS} curves at temperature varying from 250 to 350 K. (b) I_{DS}-V_{GS} curves at different temperatures. (c) Arrhenius plot of b-AsP/Au contact. (d) Extracted Schottky barrier height for b-AsP/Au contact as a function of V_{GS}, which is about 35 meV at flat-band condition.

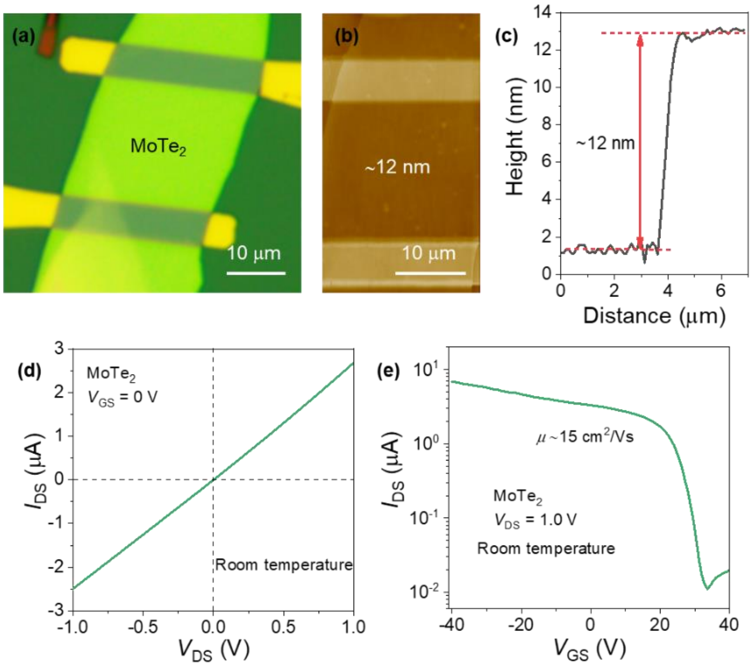

Supplementary Fig. 3 Morphology characterization and electrical properties of $MoTe_2$. (a) Optical image and (b) atomic force microscopic image of a 2D $MoTe_2$-based transistor. (c) Corresponding height profile of the $MoTe_2$ flake, showing a thickness of ~12 nm. (d) Output characteristic curve of a 2D $MoTe_2$-based transistor. Near-linear I_{DS}-V_{DS} curve was observed, indicating negligible Schottky barriers at the interface between 2D $MoTe_2$ flake and Au electrodes. (e) Transfer characteristic curve shows that the $MoTe_2$ featured a p-type behavior with a hole mobility of ~15 cm^2/Vs at room temperature.

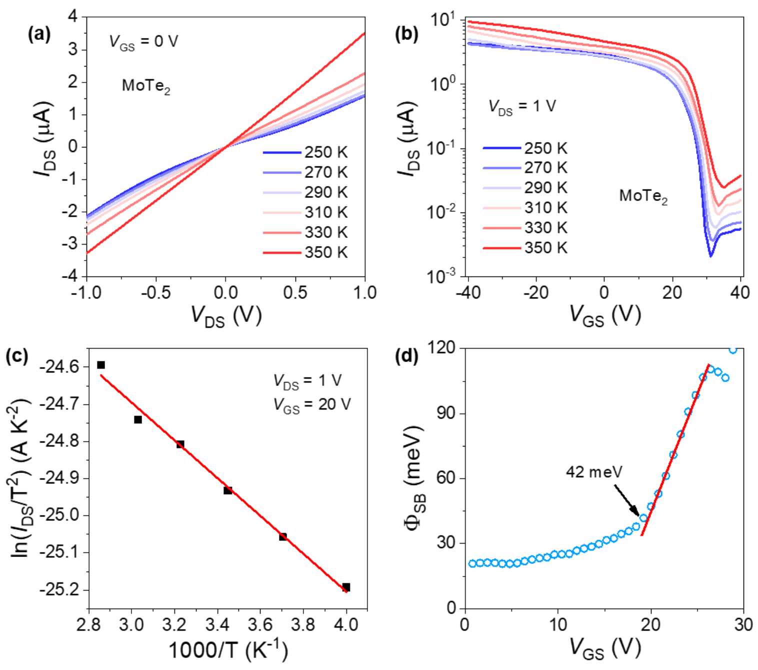

Supplementary Fig. 4 Temperature-dependent electrical characteristics of $MoTe_2$ transistor. (a) I_{DS}-V_{DS} curves at temperature varying from 250 to 350 K. (b) I_{DS}-V_{GS} curves at different temperature. (c) Arrhenius plot of $MoTe_2$/Au contact. (d) Extracted Schottky barrier height for $MoTe_2$/Au contact as a function of V_{GS}, which is about 42 meV at flat-band condition.

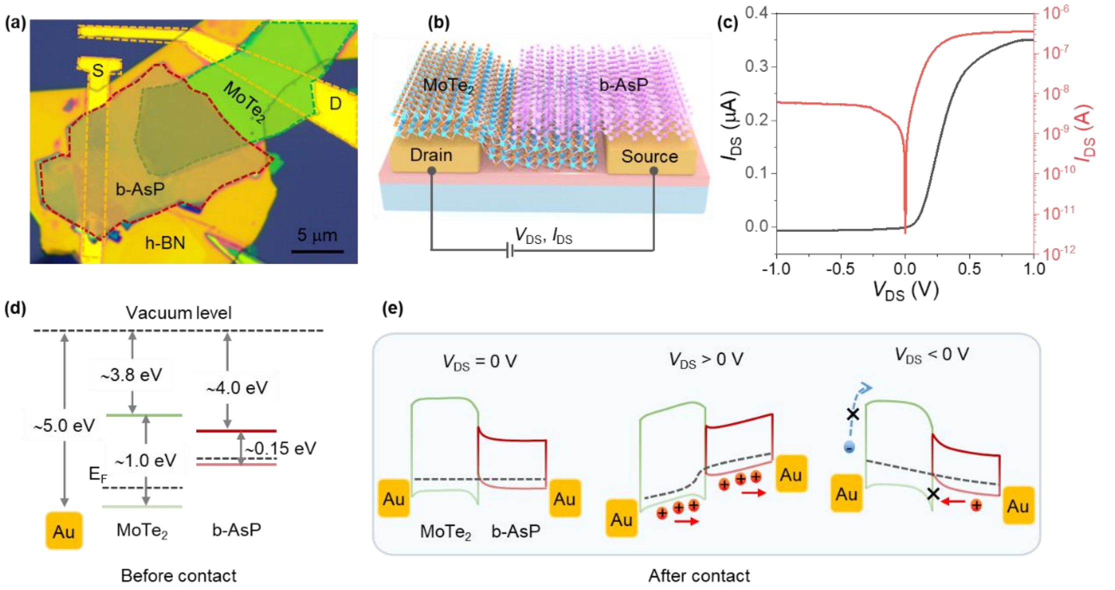

Supplementary Fig. 5 Rectification characteristic and band alignments of the b-AsP/$MoTe_2$ heterostructure. (a) Optical image of the heterostructure encapsulated by a thick h-BN flake, in which red and green dashed lines indicate the b-AsP and $MoTe_2$, respectively. (b) Schematic of the heterostructure. (c) I_{DS}-V_{DS} curve of the b-AsP/$MoTe_2$ heterostructure, showing an obvious rectification characteristic. (d) Band profiles of Au, $MoTe_2$ and b-AsP before contact. (e) Band alignments of the b-AsP/$MoTe_2$ heterostructure at different bias.

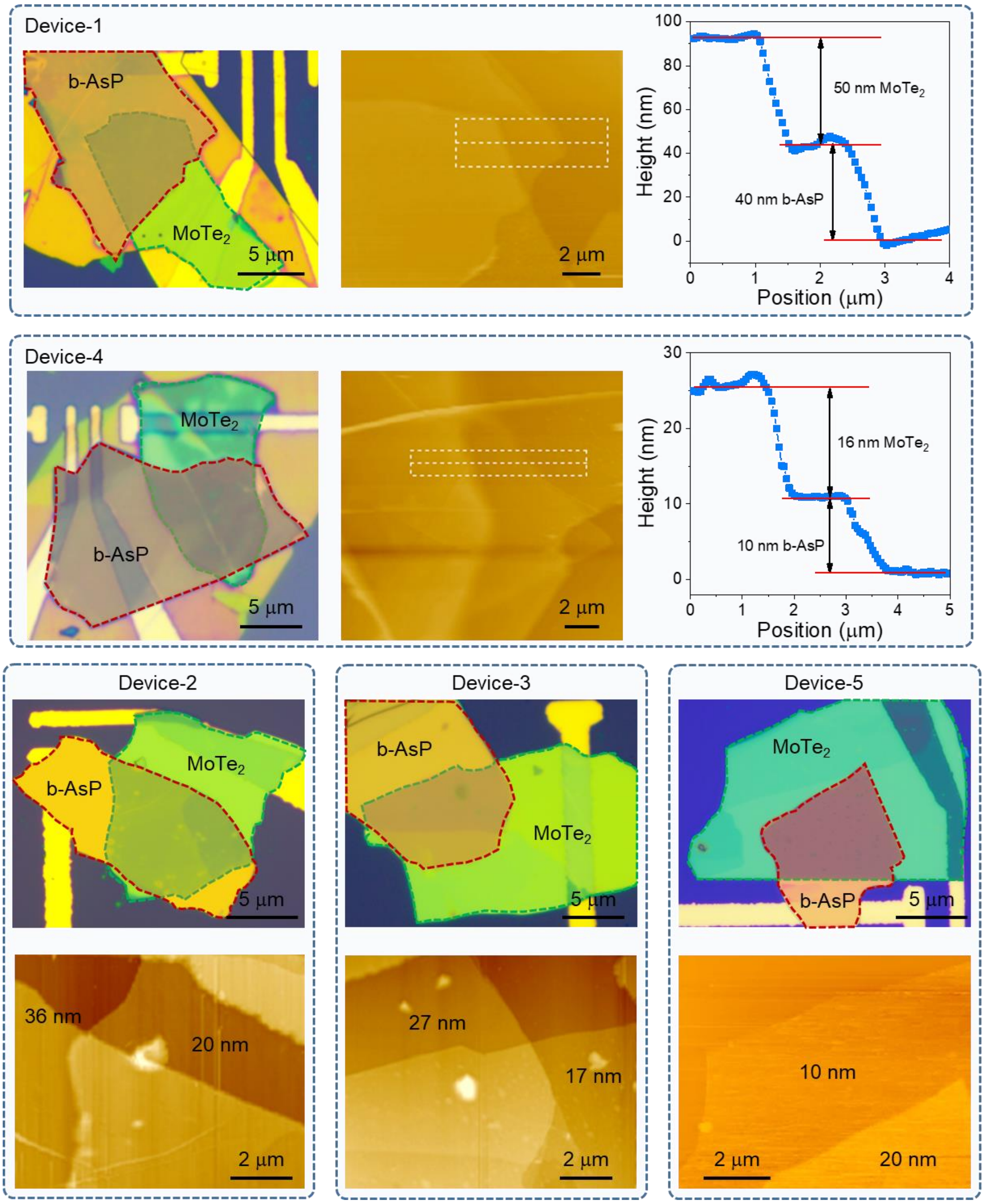

Supplementary Fig. 6 Morphology characterizations of device 1 to device 5. Note that all devices are encapsulated by h-BN flakes for perception and encoding testing.

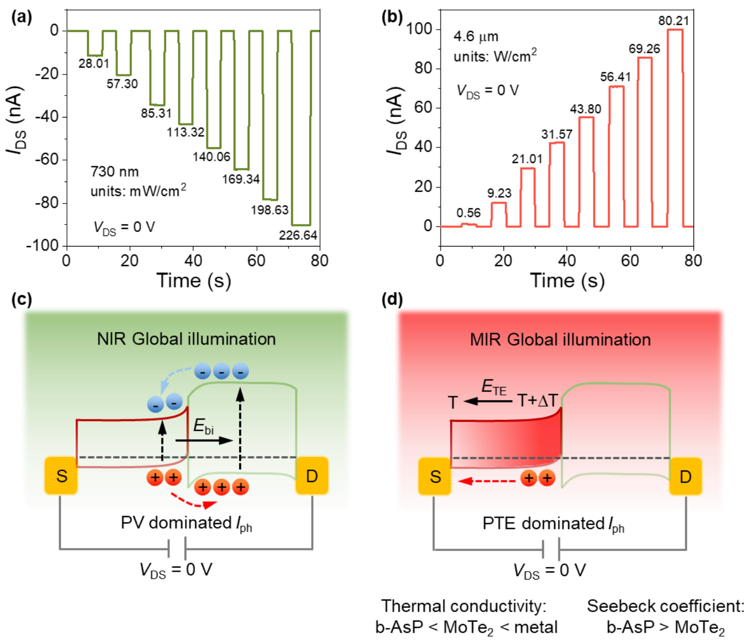

Supplementary Fig. 7 Photoresponse characterizations of device 1 at $V_{DS} = 0$ V. (a) NIR (730 nm) photoresponse with negative photocurrent at different power densities. (b) MIR (4.6 μm) photoresponse with positive photocurrent at different power densities. (c) and (d) Photoresponse mechanisms of the b-AsP/$MoTe_2$ heterostructure under NIR and MIR global illumination, respectively.

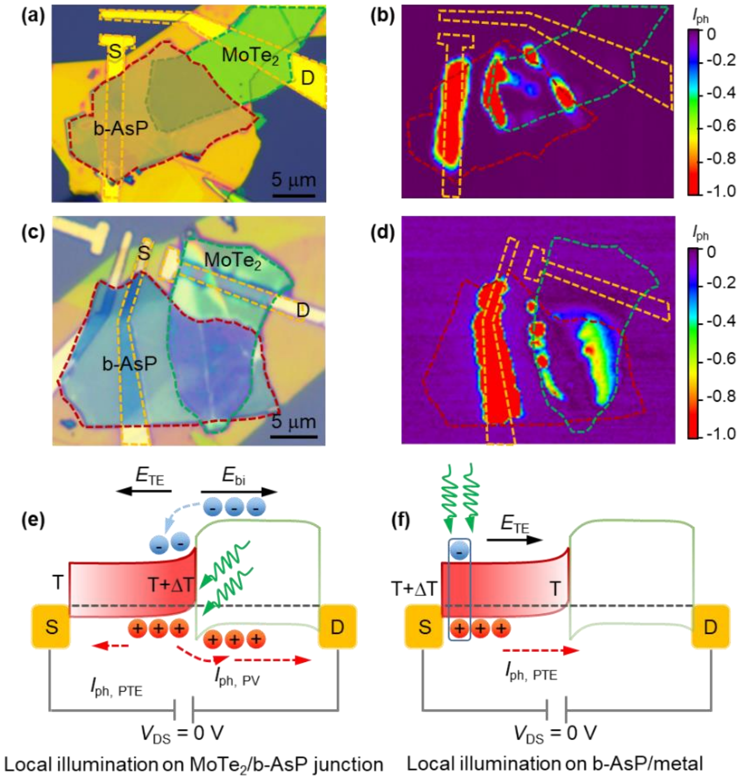

Supplementary Fig. 8 Scanning photocurrent mapping of the b-AsP/$MoTe_2$ heterostructures at V_{DS} = 0 V. (a) and (c) Optical image of b-AsP/$MoTe_2$ device 1 and 4, respectively. (b) and (d) Corresponding photocurrent mapping of device 1 and 4, respectively. The photocurrent was normalized. Note that the near-zero photocurrent in the b-AsP/$MoTe_2$ junction region may be due to the cancellation of the positive and negative photocurrent caused by PTE and PV respectively in the junction. (e) and (f) A schematic for band alignment and photo-induced carriers flow direction of the b-AsP/$MoTe_2$ heterostructures when laser locally illuminated on b-AsP/$MoTe_2$ junction and b-AsP/metal junction, respectively. The mapping images were obtained by using a focused 532 nm laser with spot of ~1 µm.

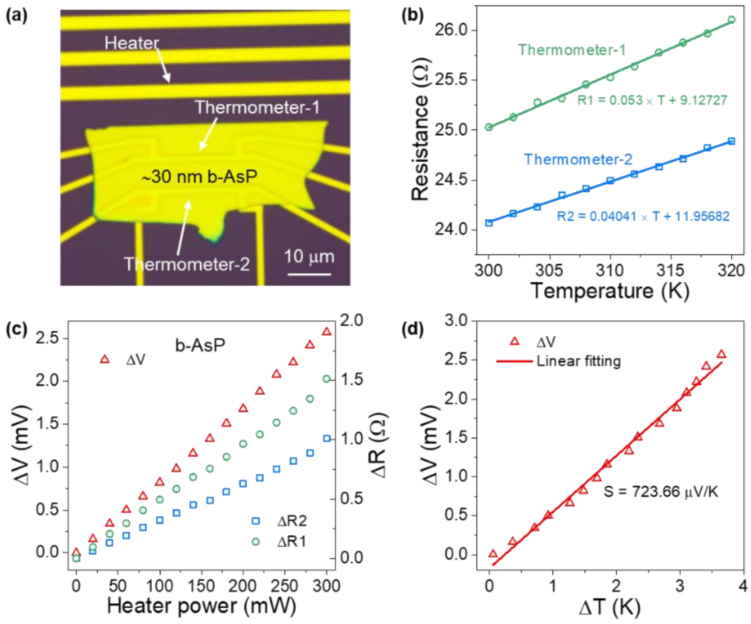

Supplementary Fig. 9 Seebeck coefficient measurement of b-AsP. (a) b-AsP device structure for thermoelectric measurement. (b) Temperature-dependent resistance of thermometer-1 and -2. (c) The voltage drop (ΔV) across b-AsP and the resistance change (ΔR) of the two thermometers at different heater powers. (d) The voltage drop (ΔV) as a function of the temperature difference between the two thermometers. The Seebeck coefficient of b-AsP was extracted to be $S = \Delta V/\Delta T = 723.66\ \mu V/K$.

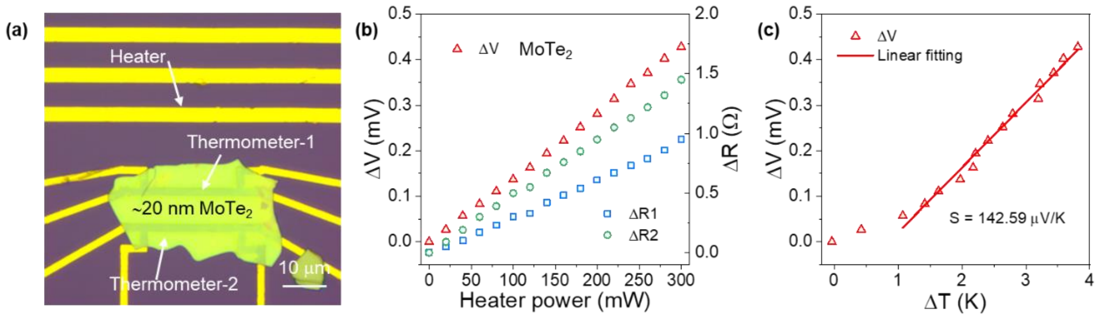

Supplementary Fig. 10 Seebeck coefficient measurement of $MoTe_2$. (a) Device structure of $MoTe_2$ for thermoelectric measurement. (b) The voltage drop (ΔV) across $MoTe_2$ and the resistance change (ΔR) of the two thermometers at different heater powers. (d) The voltage drop (ΔV) as a function of the temperature difference between the two thermometers. The Seebeck coefficient of $MoTe_2$ was extracted to be $S = \Delta V/\Delta T = 142.59\ \mu V/K$.

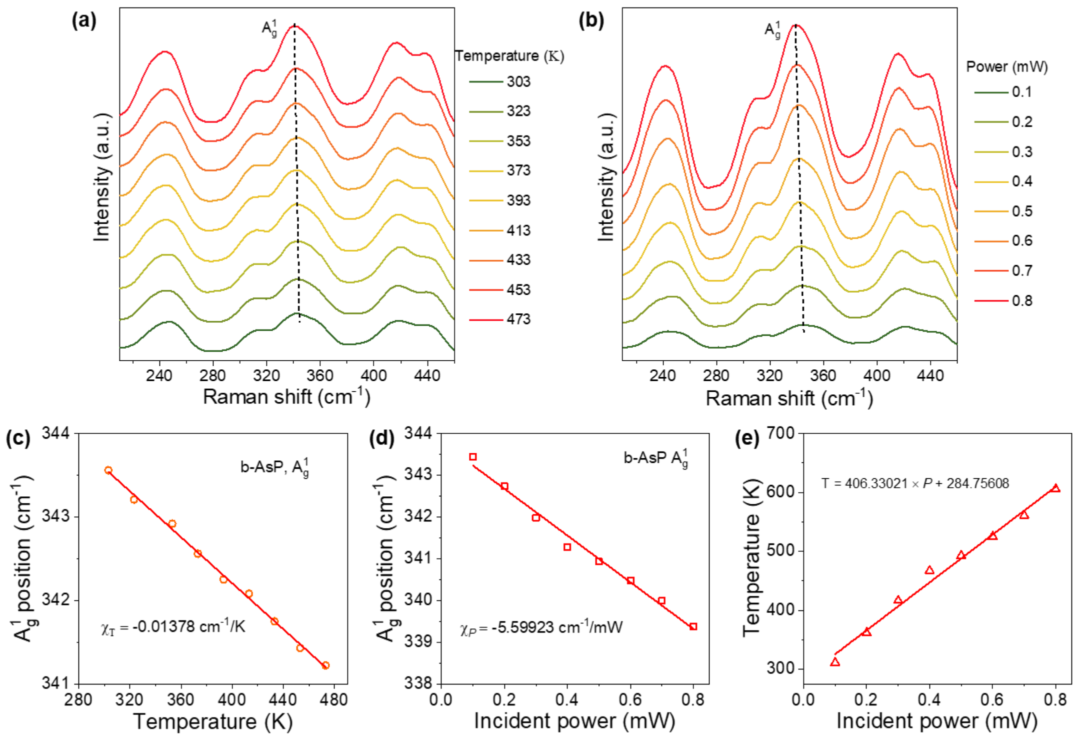

Supplementary Fig. 11 Temperature- and power-dependent Raman spectra of b-AsP. (a) Raman spectra of b-AsP at different temperatures. (b) Raman spectra of b-AsP at different power densities. (c) and (d) $A_g{}^1$ peak position as a function of temperature and incident laser power, respectively. (e) Estimated local temperature in b-AsP according to the relationship in Fig. (c) and (d), showing a linear dependence on the incident power.

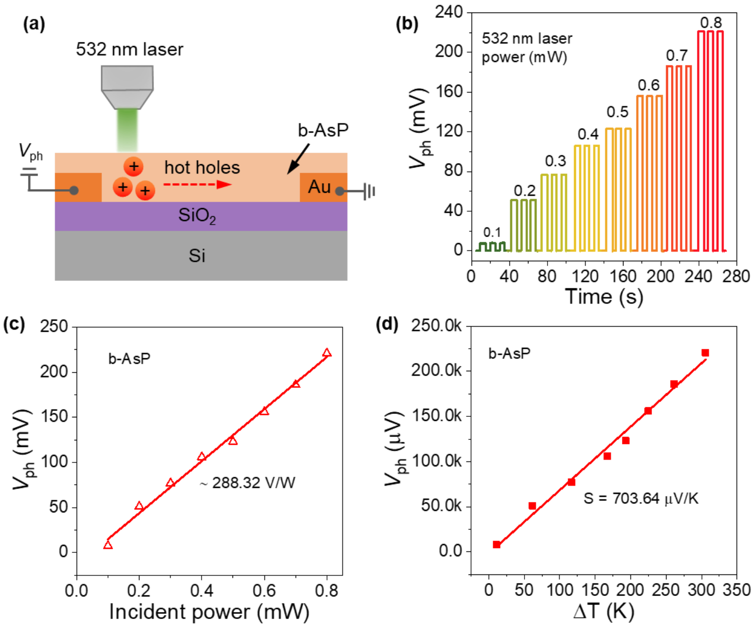

Supplementary Fig. 12 Photo-Seebeck coefficient measurement of b-AsP. (a) Schematic of photo-Seebeck coefficient measurement. A temperature gradient and a large of number of hot carriers could be induced in the b-AsP when it is locally illuminated by a focused laser. Thus, a photo-induced thermal voltage (V_{ph}) would be obtained due to the flow of hot carriers driven by the temperature gradient. (b) Time-resolved V_{ph} at different incident power. (c) V_{ph} as a function of incident power. (d) V_{ph} as a function of the temperature difference (ΔT) induced by the focused laser illumination. The temperature difference (ΔT) was extracted according to the Supplementary Fig. 11(e). The photo-Seebeck coefficient of b-AsP is calculated to be S $= V_{ph}/\Delta T = 703.64$ μV/K.

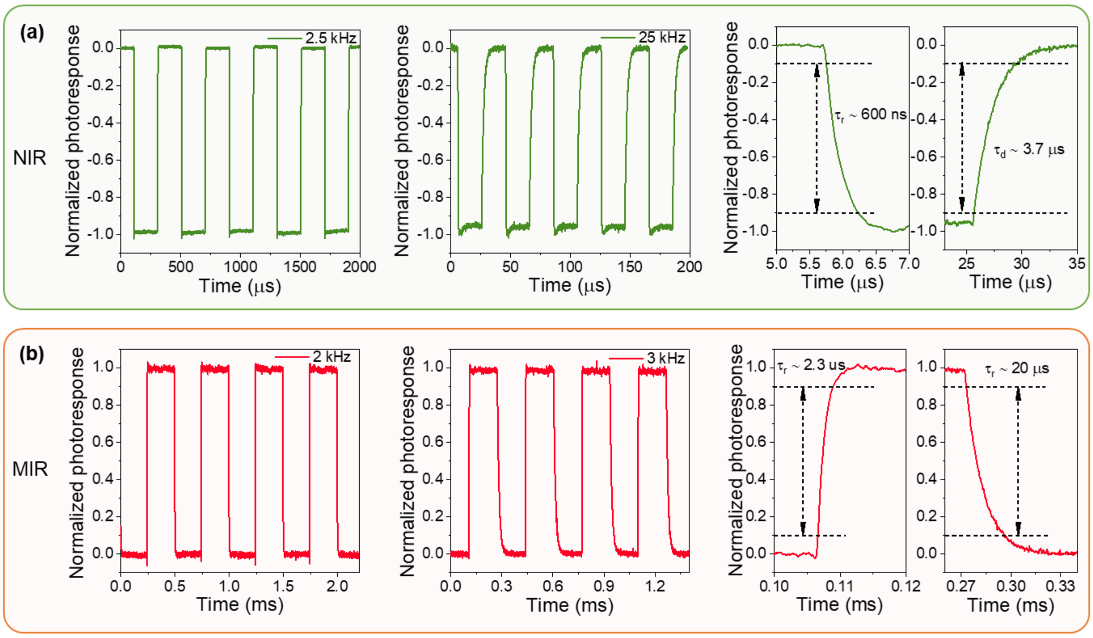

Supplementary Fig. 13 Photoresponse rate of the b-AsP/$MoTe_2$ heterostructure at V_{DS} = 0 V. (a) Transient behaviors and speed characteristics of the b-AsP/$MoTe_2$ heterostructure under 730 nm laser illumination, showing a rising time of 600 ns and a decay time of 3.7 μs. (b) MIR (4.6 μm) photoresponse rate with a rising time of 2.3 μs and a decay time of 20 μs.

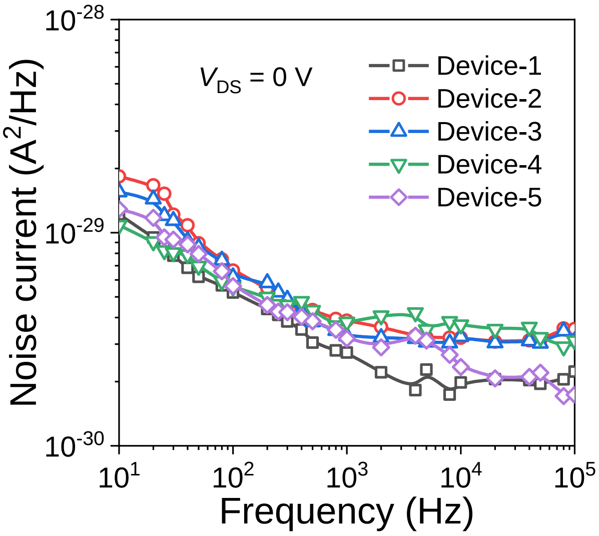

Supplementary Fig. 14 Noise characteristics of the b-AsP/$MoTe_2$ devices at V_{DS} = 0 V.

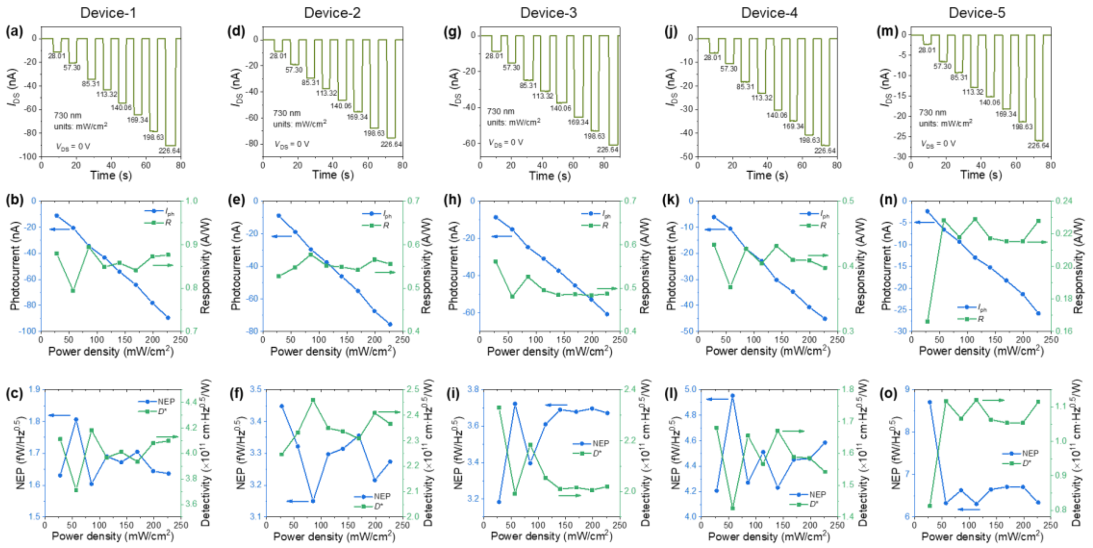

Supplementary Fig. 15 NIR (730 nm) phororesponse characteristics of the b-AsP/$MoTe_2$ heterostructures with various thicknesses at V_{DS} = 0 V. (a, d, g, j, m) Time-resolved photoresponse of the five devices under 730 nm laser illumination with various power densities. (b, e, h, k, n) Power density-dependent photocurrent and responsivity of the five devices. (c, f, i, l, o) NEP and detectivity of the five devices at different power densities.

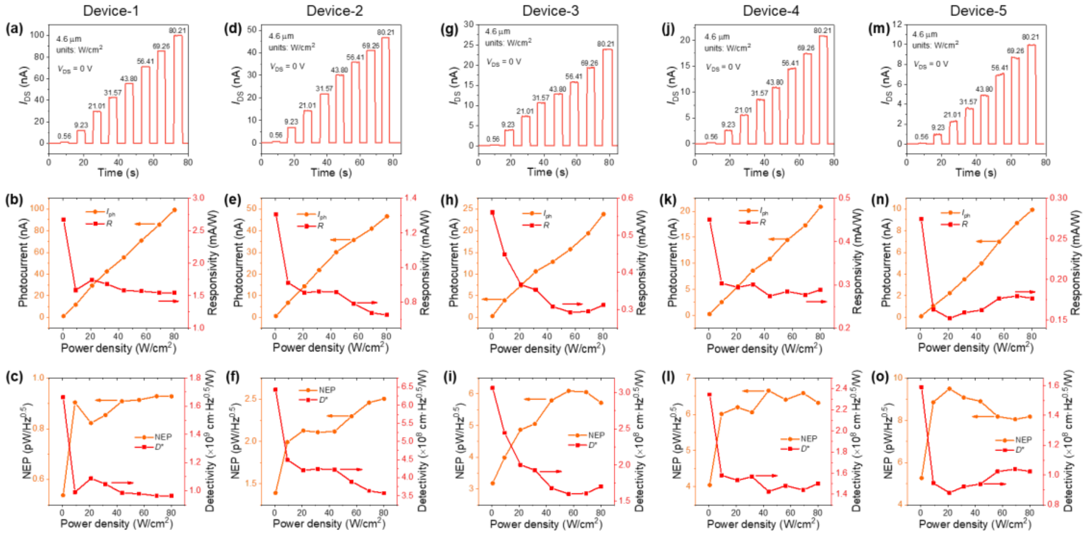

Supplementary Fig. 16 MIR (4.6 μm) phororesponse characteristics of the b-AsP/$MoTe_2$ heterostructures with various thicknesses at V_{DS} = 0 V. (a, d, g, j, m) Time-resolved photoresponse of the five devices under 4.6 μm laser illumination with various power densities. (b, e, h, k, n) Power density-dependent photocurrent and responsivity of the five devices. (c, f, i, l, o) NEP and detectivity of the five devices at different power densities.

Supplementary Table 1 Comparison of photoresponse performance of five b-AsP/$MoTe_2$ devices with various thicknesses.

Number	b-AsP Thickness (nm)	$MoTe_2$ Thickness (nm)	Junction Area (μm^2)	b-AsP Area (μm^2)	730 nm @ 226.64 mW/cm^2			4.6 μm @ 80.21 W/cm^2		
					Photocurrent (nA)	Responsivity (A/W)	Detectivity (cm·$Hz^{0.5}$/W)	Photocurrent (nA)	Responsivity (mA/W)	Detectivity (cm·$Hz^{0.5}$/W)
Device-1	~40	~50	~45	~80	-89.4	0.88	4.1×10^{11}	99.2	1.13	9.6×10^{8}
Device-2	~36	~20	~60	~80	-75.6	0.56	2.4×10^{11}	46.6	0.73	3.6×10^{8}
Device-3	~27	~17	~55	~95	-60.7	0.49	2.0×10^{11}	23.8	0.31	1.7×10^{8}
Device-4	~10	~16	~50	~90	-45.1	0.40	1.6×10^{11}	20.8	0.21	1.5×10^{8}
Device-5	~20	~10	~50	~70	-25.8	0.23	1.1×10^{11}	9.9	0.21	1.1×10^{8}

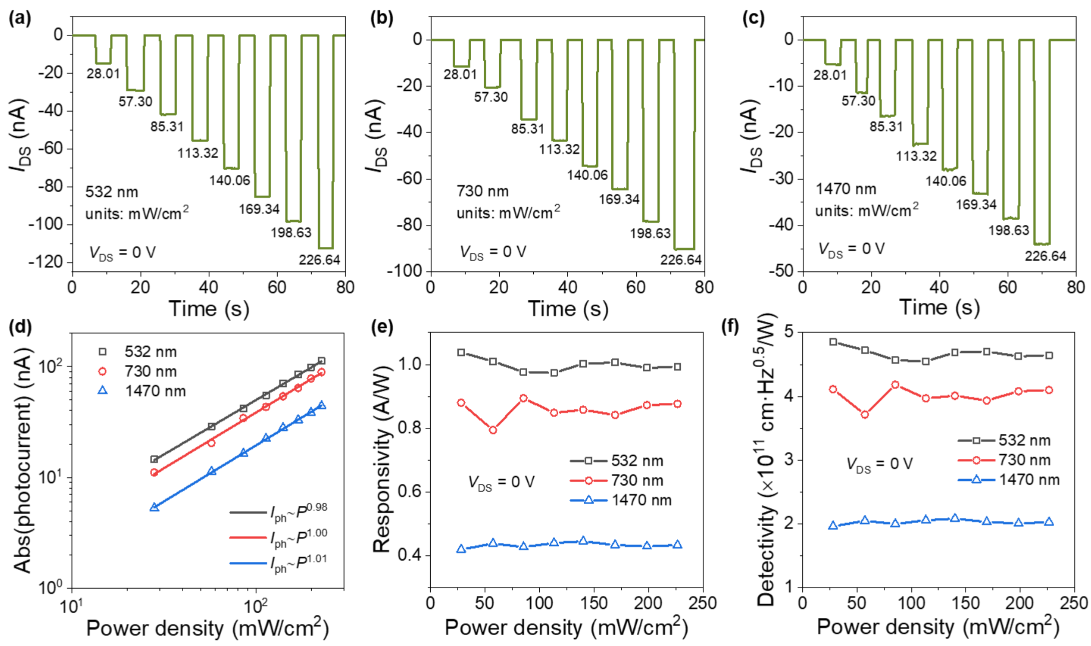

Supplementary Fig. 17 Visible/near-infrared photoresponse characteristics of the b-AsP/$MoTe_2$ heterostructure (device-1) at $V_{DS} = 0$ V. (a-c) Time-resolved photoresponse of the device under 532 nm (a), 730 nm (b), and 1470 nm (c) laser illumination with various power densities. (d-f) The power dependence of photocurrent, responsivity and detectivity of the device.

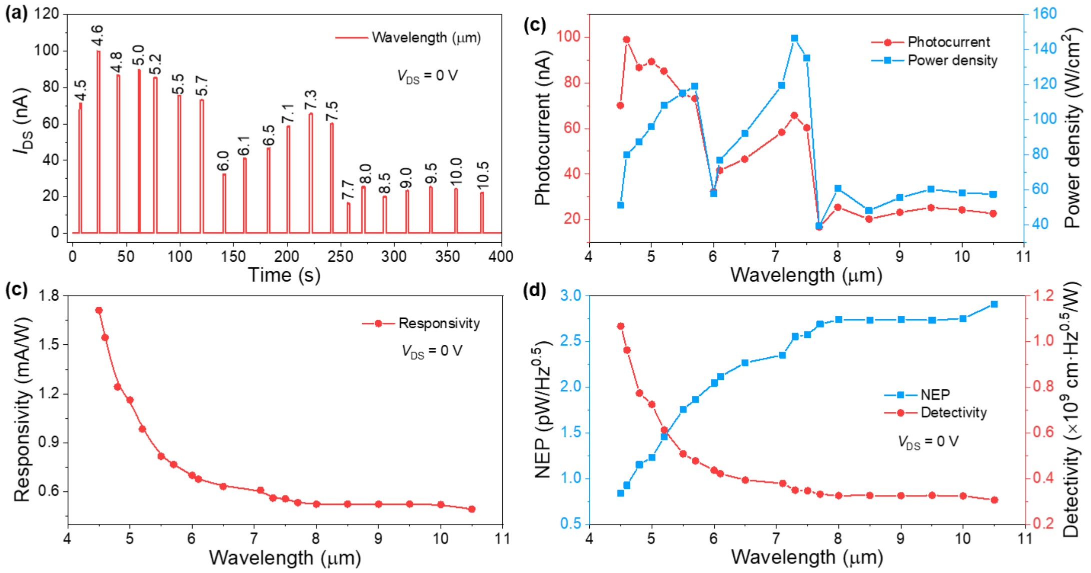

Supplementary Fig. 18 Broadband MIR photoresponse characteristics of the b-AsP/$MoTe_2$ heterostructure (device-1) at V_{DS} = 0 V. (a) Time-resolved photoresponse of the heterostructure under illumination with various wavelengths from 4.5 to 10.5 μm. (b) The photocurrent and laser power density corresponding to each wavelength. (c) Wavelength-dependent responsivity. (d) NEP and detectivity as a function of wavelength.

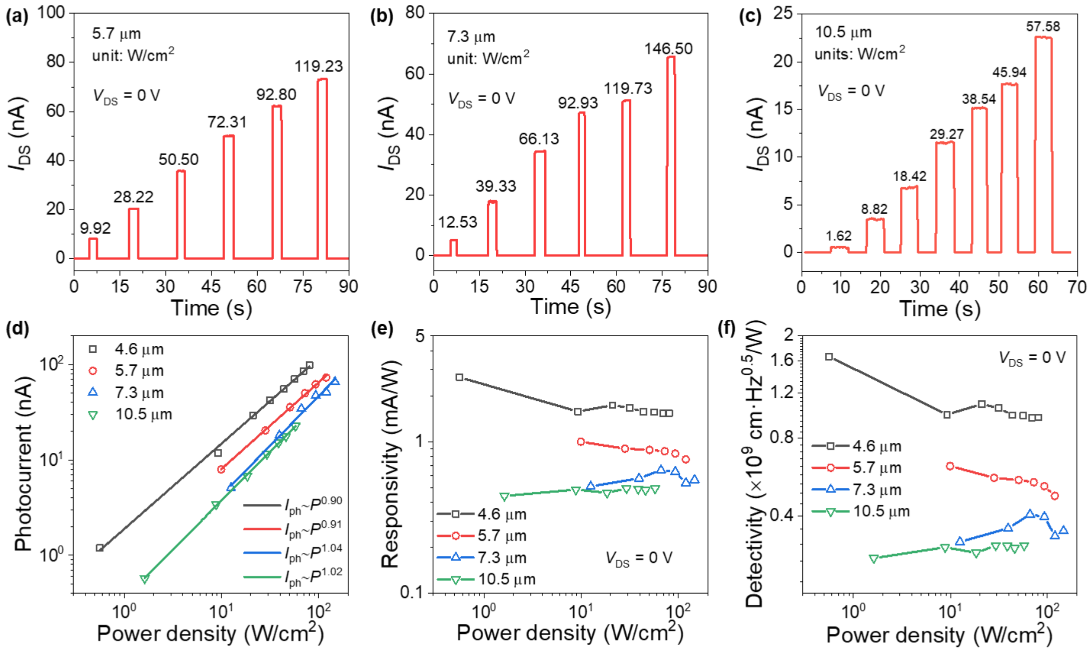

Supplementary Fig. 19 MIR photoresponse characteristics of the b-AsP/$MoTe_2$ heterostructure (device-1) at V_{DS} = 0 V. (a-c) Time-resolved photoresponse of the device under 5.7 μm (a), 7.3 μm (b), and 10.5 μm (c) laser illumination with various power densities. (d-f) The power dependence of photocurrent, responsivity and detectivity of the device.

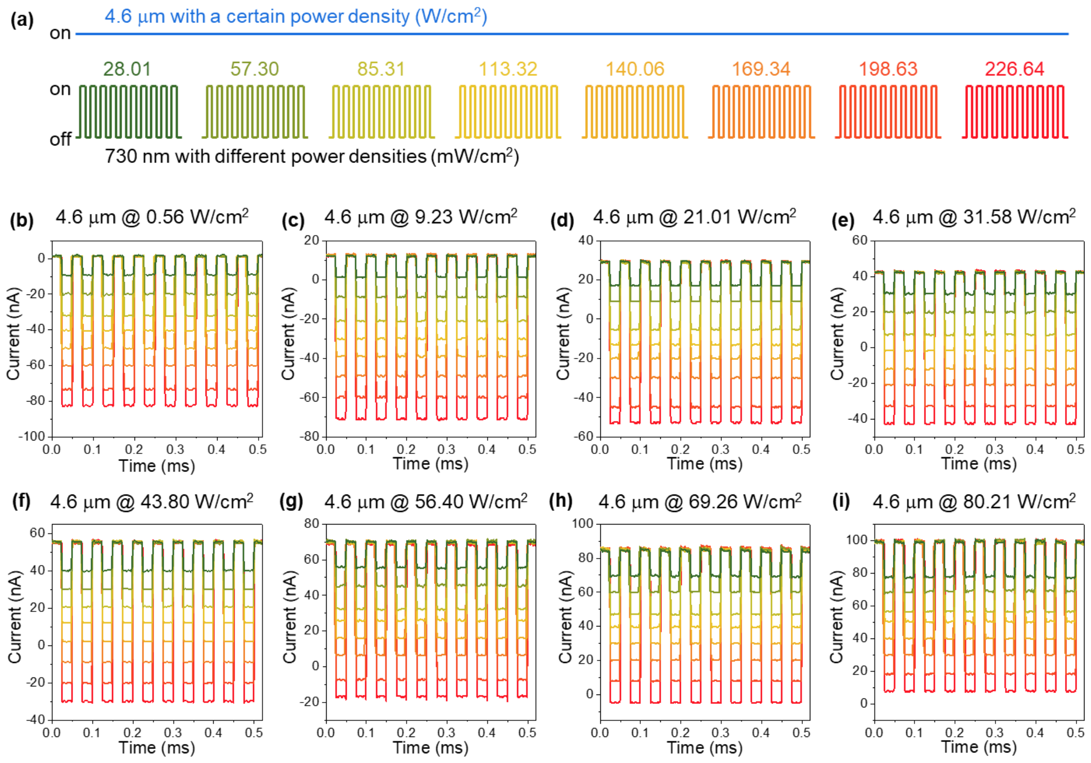

Supplementary Fig. 20 Phororesponse of the b-AsP/$MoTe_2$ heterostructure (device-1) under simultaneous illuminations of both 4.6 μm and 730 nm with various power densities. (a) Measurement conditions, namely the heterostructure was simultaneously illuminated by 4.6 μm laser with a certain power density and 730 nm laser with various power densities. (b-i) Corresponding photoswitching response. Note that 730 nm is irradiated to the heterostructure in the form of pulses at a frequency of 20 kHz.

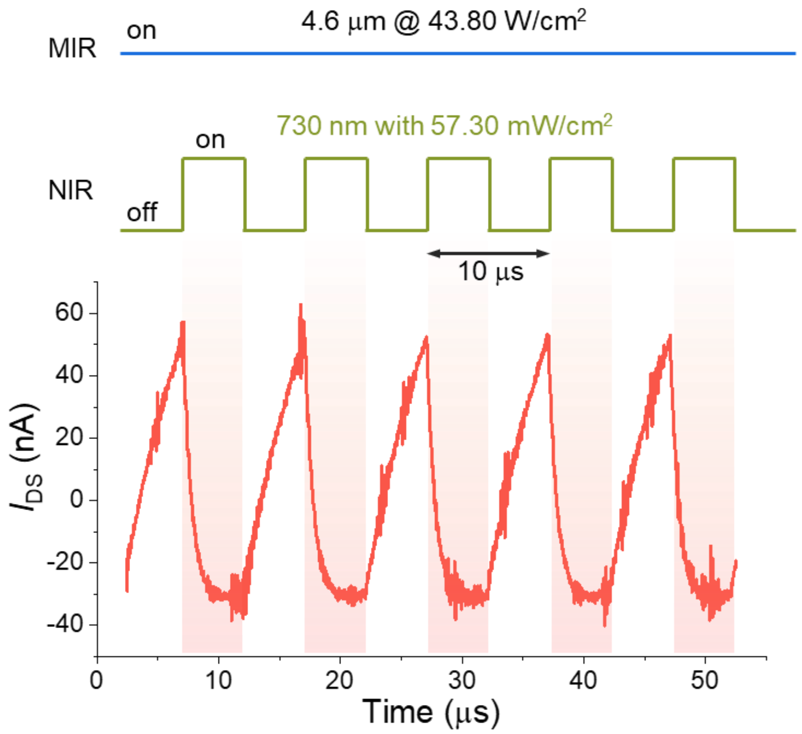

Supplementary Fig. 21 Fast phororesponse of the b-AsP/$MoTe_2$ heterostructure (device-1) under simultaneous illuminations of both 4.6 μm and 730 nm.

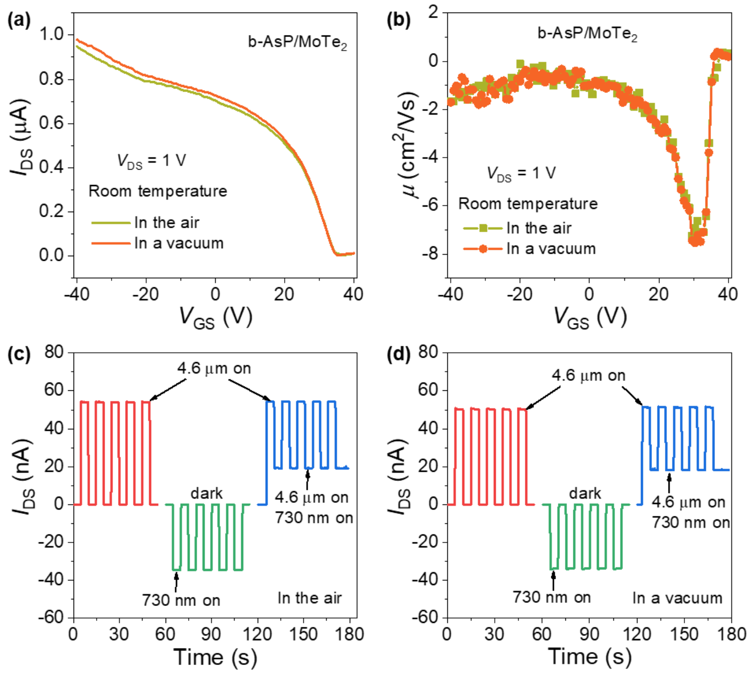

Supplementary Fig. 22 The comparison of carrier mobility and photoresponse characteristics of the b-AsP/$MoTe_2$ device in the air and a vacuum (~10^{-6} Torr). (a) I_{DS}-V_{GS} curves of the device in the air and a vacuum at room temperature. (b) As-calculated carrier mobility from (a), showing a negligible change from the air to a vacuum. (c) and (d) Photoresponse characteristics of the device at V_{DS} = 0 V in the air and a vacuum, respectively. The almost unchanged carrier mobility and photoresponse indicates that the h-BN-encapsulated b-AsP/$MoTe_2$ device possesses good stability.

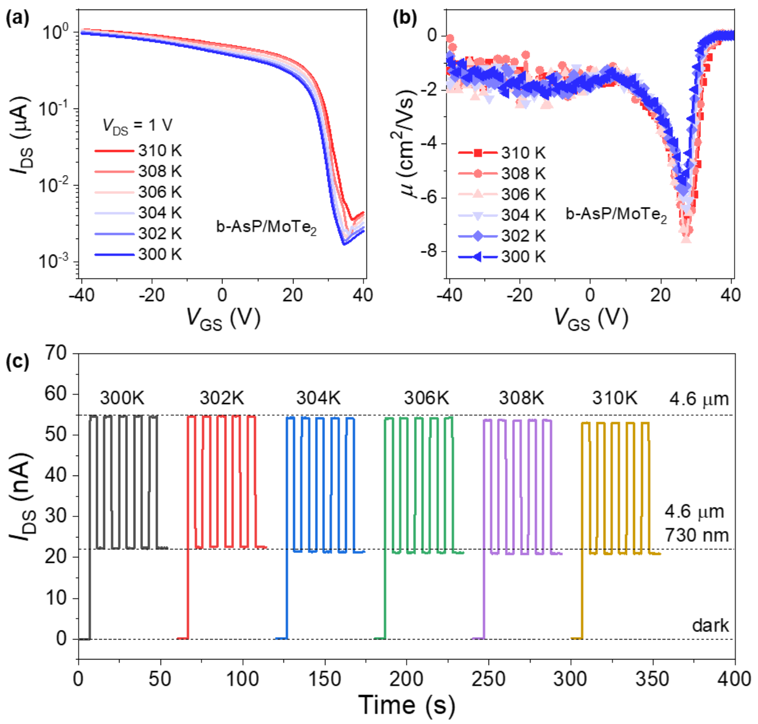

Supplementary Fig. 23 The effect of temperature variation on the carrier mobility and photoresponse characteristics of the b-AsP/$MoTe_2$ device. (a) I_{DS}-V_{GS} curves of the device at various temperatures from 300 to 310 K. (b) As-calculated carrier mobility from (a), showing little change from 300 to 310 K. (c) Photoresponse characteristics of the device at different temperatures, showing a slight decrease (<2 nA) with increasing the temperature from 300 to 310 K. Such a small effect is not enough to affect device performance.

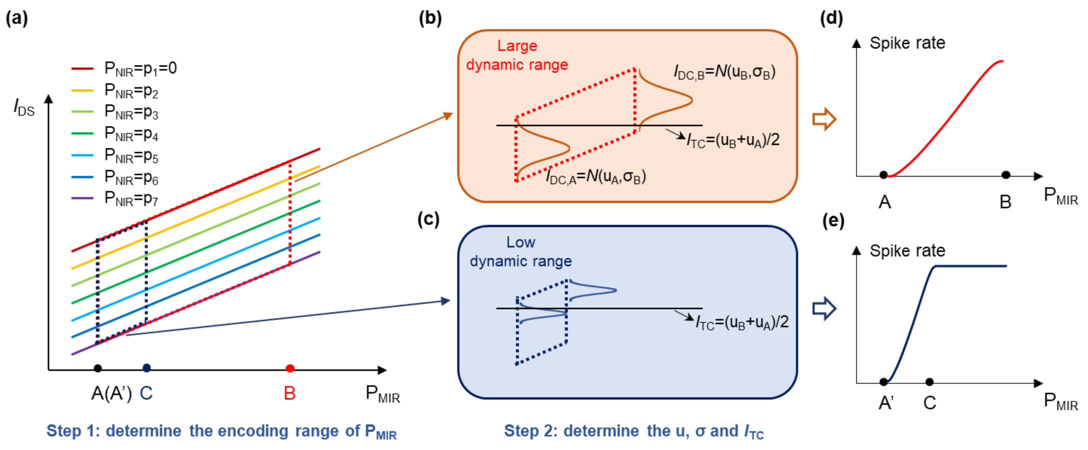

Supplementary Fig. 24 Schematic diagram about how to determine the suitable encoding parameters (mean (u), variance (σ) and spiking threshold current (I_{TC})) to realize high encoding precision. (a) The schematic of the I_{DS} as the function of P_{MIR} and P_{NIR}. The used optical power of NIR light (P_{NIR}) ranges from 0 to P_7. The first step is to determine the encoding dynamic range of P_{MIR}. Two encoding dynamic ranges of P_{MIR}, [A, B] and [A', C], are marked by dashed line. (b, c) Schematic of explaining how to determine the u, σ and I_{TC}. (d, e) The corresponding encoding transfer curves using parameters in (b, c).

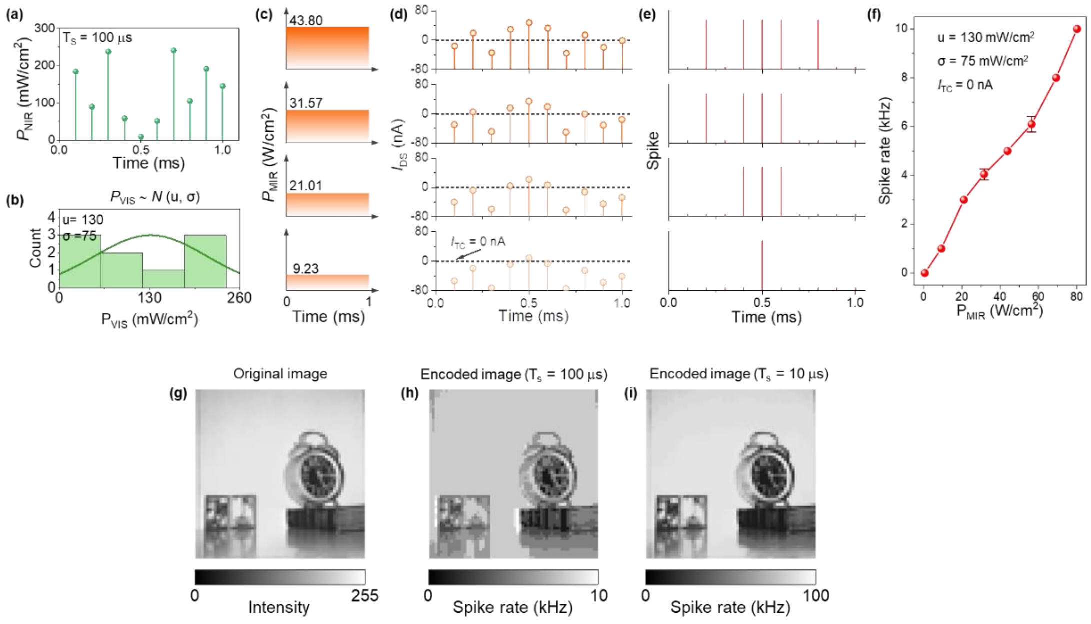

Supplementary Fig. 25 The experimental and simulation results when sampling period (T_S) for NIR light is 100 µs. (a) One trail of the NIR optical pulses that are randomly sampled from a Gaussian distribution with u = 130 mW/cm^2 and σ = 75 mW/cm^2. The time-steps are 10 given T_S = 100 µs under a total encoding time of 1 ms. (b) The fitting distribution from the trail of NIR optical pulses. (c) Analog value of MIR power density. (d) Corresponding time-domain transduction current (I_{DS}) waveform output from the source electrode for each MIR power density in (c). (e) Corresponding spike train for each I_{DS} waveform in (d) when the spike threshold current (I_{TC}) is set to 0 nA. The I_{DS} higher than I_{TC} could stimulate one spike. (f) Mean spike rate as a function of P_{MIR} when u, σ, I_{TC} are 130 mW/cm^2, 75 mW/cm^2, 0 nA, respectively. Error bars in (f) represent the variation (standard deviation) of spike rate. (g) The original clock image. The pixel values ranging from 0 to 255 are linearly mapped to MIR optical power density of 0 to 80.21 W/cm^2. (h) The encoded clock image at T_S of 100 µs. The time-steps for encoding each pixel are 10. The maximal spike rate is 10 kHz. When T_S decreases to 10 µs, (i) shows the encoded clock image with maximal spike rate of 100 kHz.

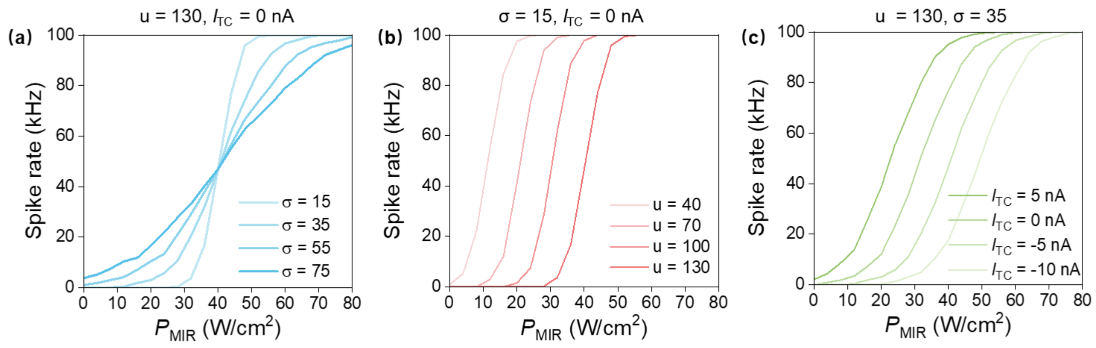

Supplementary Fig. 26 (a-c) Simulation of spike rate as a function of P_{MIR} for different u, σ and I_{TC} of the Gaussian distribution used for sampling 730 nm light.

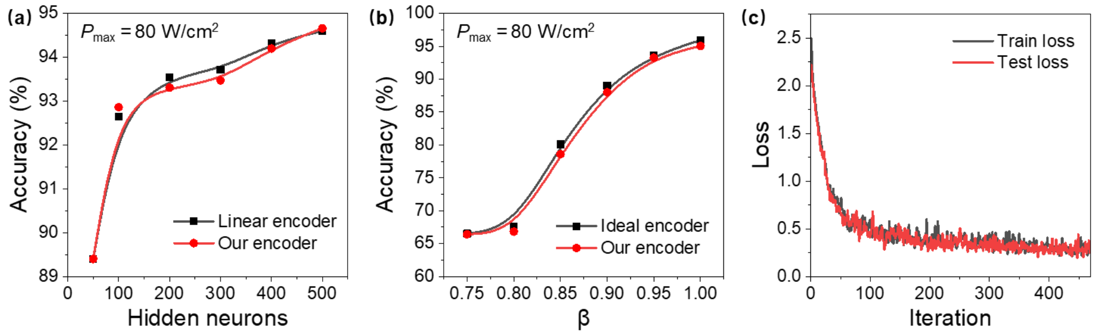

Supplementary Fig. 27 Classification accuracy versus (a) hidden neurons and (b) β for data set with $P_{\max}$ of 80.21 W/cm^2. (c) Cross-entropy loss of SNN versus iteration for train and test set.

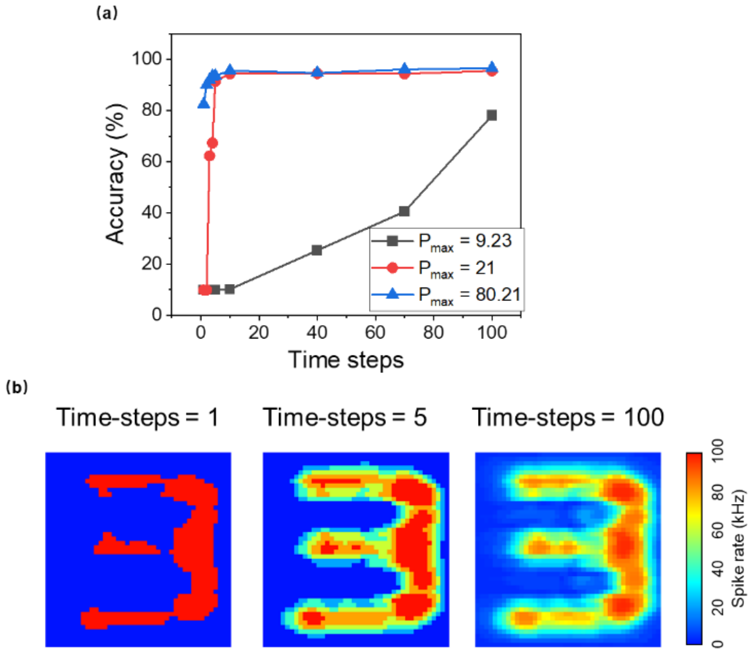

Supplementary Fig. 28 (a) The inference accuracy of the SNN when the device uses different time-steps for encoding MIR objects with different P_{max}. (b) The encoded images of MIR target with P_{max} = 80.21 W/cm^2 at time-steps of 1, 5, 100. The results show that MIR objects with lower optical power requires more time-steps for accurate encoding and recognition. That means high-speed response of the device to NIR light is critical for accurate and fast MIR objects classification under a certain constrain in encoding time.

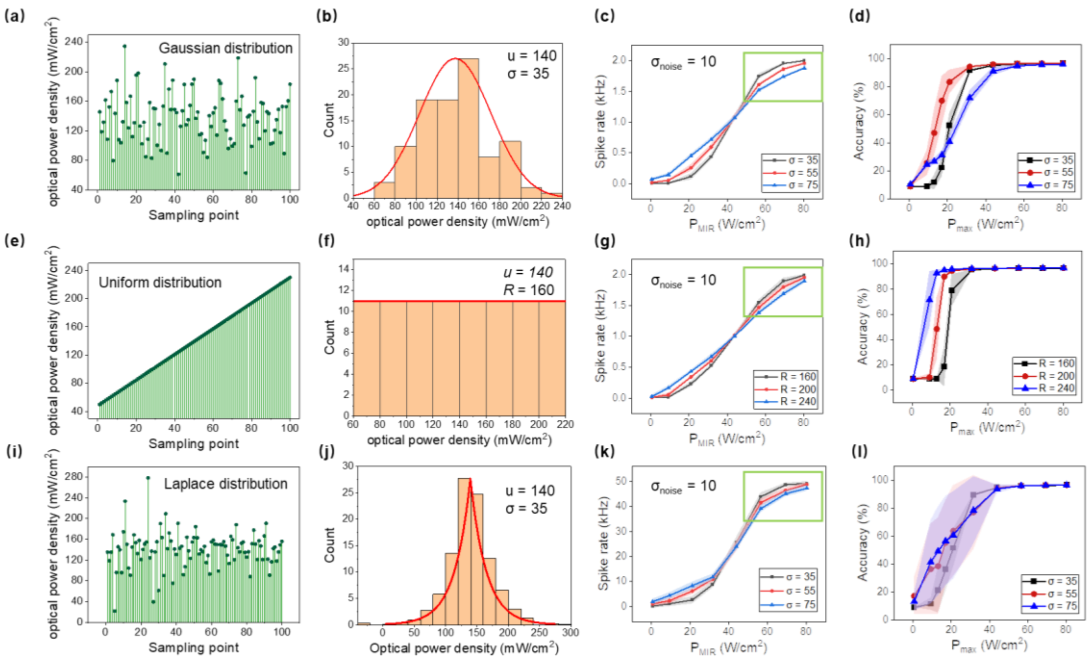

Supplementary Fig. 29 The impact of different distributions for sampling NIR light on the encoding precision and image recognition accuracy. The Gaussian, Uniform and Laplace distributions are discussed here. The white noise with variance (σ_{noise}) of 10 mW/cm^2 is introduced to the simulation to investigate the noise-tolerance of different distribution. The sampling sequence of NIR optical power density, the distribution fitting curve, the encoding transfer curve, and the recognition accuracy for (a-d) Gaussian distribtion with u = 140 mW/cm^2 and σ = 35 mW/cm^2. (e-h) Uniform distribution for u = 140 mW/cm^2 and optical power density range for sampling NIR light (R) of 160 mW/cm^2. (i-l) Laplace distribution with u = 140 mW/cm^2 and σ = 35 mW/cm^2. The recognition accuracy variations (standard deviations) of different distributions are shown as the shaded area in (d), (h) and (i).

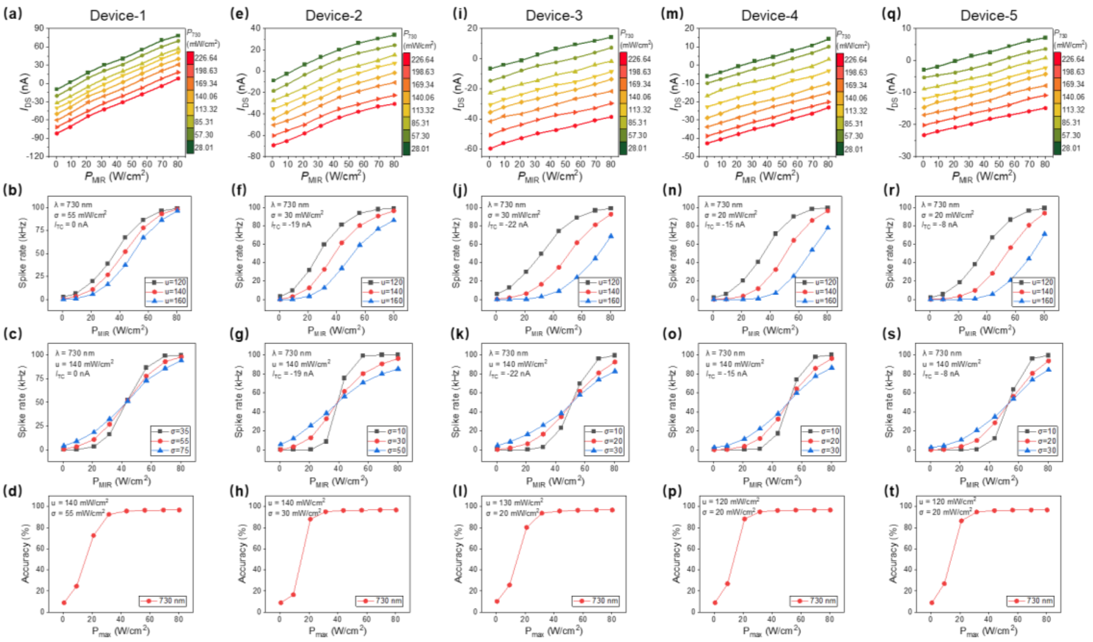

Supplementary Fig. 30 The photocurrent response, encoding transfer curve and recognition accuracy towards MIR-MNIST datasets of five devices with different thickness. Device 1: (a-d); Device 2: (e-h); Device 3: (i-l); Device 4: (m-p); Device 5: (q-t).

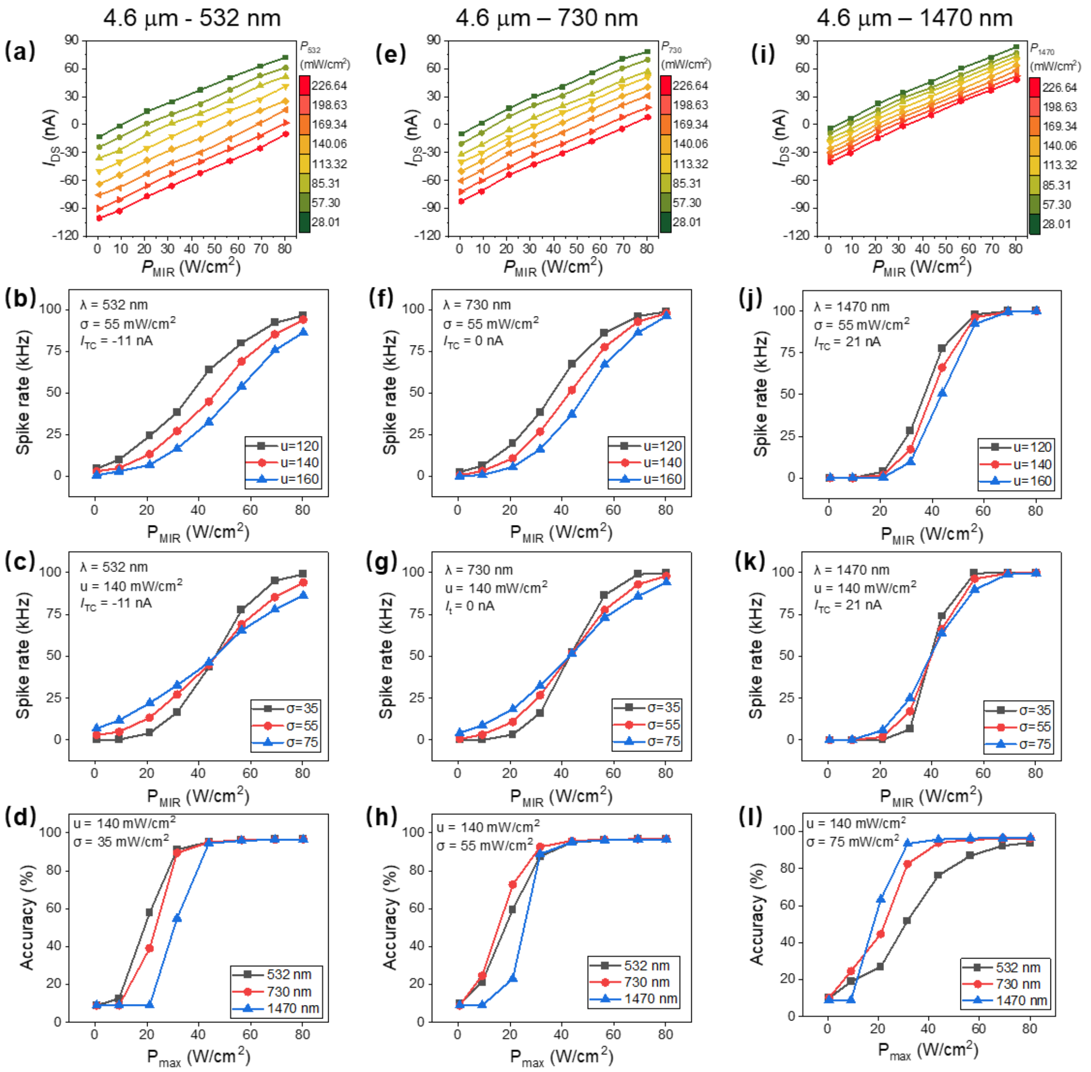

Supplementary Fig. 31 The photocurrent response and the encoding transfer curve under different σ and u when using (a-c) 532 nm, (e-g) 730 nm and (i-k) 1470 nm laser as the stochastic sampling light. (d-l) The recognition accuracy of the device for MIR-MNIST data set at different σ when using three different wavelengths

Supplementary Note 1. Electrical characterizations of the b-AsP/$MoTe_2$ heterostructure

The carrier mobility and contact barrier of individual b-AsP and $MoTe_2$ are evaluated, as shown in Supplementary Fig. 1-4. The nearly linear I_{DS}-V_{DS} curves in Supplementary Fig. 1d and 3d suggest that good contacts are formed between the b-AsP ($MoTe_2$) and Au electrodes. According to the I_{DS}-V_{GS} curves shown in Supplementary Fig. 1e and 3e, both b-AsP and $MoTe_2$ exhibit p-type conduction behavior. Their hole-dominated carrier mobilities (μ) are calculated using the relation:

$$\mu = \frac{dI_{DS}}{dV_{GS}} \frac{L}{WV_{DS}C_{ox}} \quad (1)$$

Where L and W are the length and width of the channel, respectively, I_{DS}, V_{DS} and V_{GS} refer to the source-drain current, bias voltage and gate voltage, respectively, and C_{ox} is the dielectric oxide capacitance (13.4 nF/cm^2 for our used SiO_2). The as-calculated hole mobility of the b-AsP and $MoTe_2$ are reach up to ~145 and ~15 cm^2/Vs, which is consistence with previous reports.[1,2] The contact barriers (Schottky barriers) between b-AsP ($MoTe_2$) and Au electrodes are extracted by temperature-dependent electrical characteristics, which are presented in Supplementary Fig. 2 and 4. The extraction of Schottky barrier is based on a thermionic model by using Arrhenius plots with the following equation:[3]

$$\ln\left(\frac{I_{DS}}{T^2}\right) = -\frac{\Phi_{SB}}{kT} + c \quad (2)$$

where I_{DS} is the source-drain current, T is temperature, Φ_{SB} is the Schottky barrier height, k is the Boltzmann constant and c is a constant. As shown in Supplementary Fig. 2d and 4d, the as-extracted Φ_{SB} for b-AsP/Au and $MoTe_2$/Au junction are 35 meV and 42 meV under the flat-band condition, respectively. We note that the individual b-AsP, $MoTe_2$ and b-AsP/$MoTe_2$ heterostructure devices are fabricated by dry transferring the exfoliated b-AsP and $MoTe_2$ flakes onto the prefabricated Au electrodes. Therefore, such small Schottky barriers may be attributed to the nondestructive contact form.[3] The good contacts and high carrier mobility enable the b-AsP/$MoTe_2$ heterostructure to have excellent photoresponse performance.

The optical image and device configuration of an as-fabricated b-AsP/$MoTe_2$

heterostructure (device 1) are shown in Supplementary Fig. 5a-b, where b-AsP and $MoTe_2$ are marked by red and green dashed line, respectively. More b-AsP/$MoTe_2$ heterostructures (devices 1-5) with various thicknesses are presented in Supplementary Fig. 6. Note that all devices are finally encapsulated by insulating h-BN flakes to prevent them from being corroded by water and oxygen in the air. The I_{DS}-V_{DS} curve (Supplementary Fig. 5c) of the b-AsP/$MoTe_2$ heterostructure shows diode-like rectification characteristics, indicating the existence of built-in electric field in the overlap region. Note that the rectification characteristics induced by metal-semiconductors junction could be neglected due to their small Schottky barrier heights (Supplementary Fig. 2d and 4d). Supplementary Fig. 5d and 5e depict the band alignments of the b-AsP/$MoTe_2$ heterostructure before and after contact, in which the energy level positions are obtained from the references.[4,5] Based on the band alignments, the diode-like rectification characteristics can be easily understood. In equilibrium state (V_{DS} = 0 V), the minority electrons in b-AsP would move into $MoTe_2$, thereby causing a wider depletion layer at $MoTe_2$ side and creating more holes at b-AsP side. In other words, a built-in electric field with direction pointing to $MoTe_2$ side is formed at the interface of the b-AsP/$MoTe_2$ heterostructure. At positive bias (V_{DS} > 0 V), the majority holes in $MoTe_2$ and minority electrons in b-AsP easily cross over the interface barrier, forming a large current. At negative bias (V_{DS} < 0 V), the electrons can hardly be injected into $MoTe_2$ due to the high Schottky barrier for electrons, while the holes in b-AsP could cross the interface barrier due to the small valance band offset (~0.2 eV). Even so, the current at negative bias is lower than that at positive bias.

Supplementary Note 2. Photoresponse characteristics of the b-AsP/$MoTe_2$ heterostructure

The laser spots of MIR (4.6 μm) laser and NIR (730 nm) laser are about 100 μm, which is larger than the size scaling of the as-fabricated 2D b-AsP/$MoTe_2$ heterostructures. Thus, the entire device can be considered to be uniformly illuminated. As shown in Supplementary Fig. 7a-b, the b-AsP/$MoTe_2$ heterostructure (device 1) shows negative and positive photoresponse under 730 nm and 4.6 μm laser illumination, respectively. The same photoresponse behaviors have been observed in other b-AsP/$MoTe_2$ devices with various thicknesses (Supplementary Fig. 15 and 16). Moreover, the negative photoresponse behavior also can be observed by using 532 nm and 1470 nm laser illumination (Supplementary Fig. 17), and the positive photoresponse behavior also can be observed by applying other MIR lasers with wavelengths ranging from 4.5 μm to 10.5 μm (Supplementary Fig. 18 and 19). For the illumination, the visible/NIR light could be absorbed by both $MoTe_2$ and b-AsP, while the MIR absorption mainly occurs in b-AsP, because the MIR absorption of $MoTe_2$ can be ignored due to its large bandgap of ~1.0 eV. In addition, the thermal conductivity of b-AsP (~33 W/mK)[6] and $MoTe_2$ (~40 W/mK)[7,8] is less than that of Au electrode (~200 W/mK),[9] and the Seebeck coefficient of b-AsP (~803 μV/K)[10] is higher than that of $MoTe_2$ (~230 μV/K).[1] In view of these, we preliminarily believe that the negative photoresponse under visible and NIR illumination is caused by the photovoltaic (PV) effect driven by the built-in electrical filed of b-AsP/$MoTe_2$ junction, while the positive photoresponse is induced by the photothermoelectric (PTE) effect in b-AsP.[11] More experiments are performed to support the above points, which are described as follows:

First, we measured the Seebeck coefficient of b-AsP by fabricating a thermoelectric device. As shown in Supplementary Fig. 9, by applying a certain voltage to the heater, the heating power is injected into the device, and a temperature gradient (ΔT) across the b-AsP is created. The local temperature at both ends of b-AsP could be read out by the pre-calibrated thermometer-1 and thermometer-2. Simultaneously, the thermoelectric voltage (ΔV) induced by temperature gradient was measured by

thermometer-1/2. The thermoelectric voltage (ΔV) is plotted against the temperature gradient (ΔT) with a linear trend, from whose slope we extract the Seebeck coefficient of b-AsP to be S = ΔV/ΔT = 723.66 μV/K. In the same way, the Seebeck coefficient of $MoTe_2$ was measured to be 142.59 μV/K (Supplementary Fig. 10). In addition, we also evaluated the photo-seebeck coefficient of b-AsP by combing temperature-dependent and power-dependent Raman spectra as well as local illumination induced photo-voltage measurements. As shown in Supplementary Fig. 11 and 12, the photo-Seebeck coefficient of b-AsP was evaluated to be 703.64 μV/K, which is close to the value measured by thermoelectric device.

Second, we calculated the Schottky barrier height of b-AsP/Au and $MoTe_2$/Au contacts via temperature-dependent electrical characteristics (Supplementary Fig. 2 and 4), which are extracted to be 35 meV and 42 meV, respectively. Although there are asymmetric Schottky barriers between b-AsP/Au and $MoTe_2$/Au, such small barrier heights and barrier differences are difficult to contribute significant photocurrent.

Third, we carried out scanning photocurrent mapping to distinguish the photocurrent generation locations by using a focused 532 nm laser with a laser spot of ~1 μm, as shown in Supplementary Fig. 8. It is can be observed that the photocurrents mainly originated from the junction region and b-AsP on the Au electrode, while there is no obvious photocurrent in the $MoTe_2$/Au region even though its contact barrier is higher than that of b-AsP/Au. Therefore, we believe that Schottky photovoltaic effect in metal/semiconductors junction can be neglected in our device. The photocurrent generated in the b-AsP/$MoTe_2$ junction region should be contributed to the photovoltaic effect. When the laser is illuminated on the b-AsP/$MoTe_2$ junction region, electron-hole pairs are generated in both b-AsP and $MoTe_2$. Then, the photo-generated electrons and holes are driven to b-AsP (source electrode) and $MoTe_2$ (drain electrode) under the built-in field, respectively, thus resulting in negative photocurrent. As for the photocurrent in the b-AsP/Au junction, we believe it is caused by photothermoelectric effect (PTE) of b-AsP. As mentioned above, b-AsP possesses a high Seebeck coefficient, which enables a significant temperature gradient from the b-AsP/Au to b-AsP/$MoTe_2$ when the laser locally illuminated on the b-AsP/Au junction. Thus, the hot holes could

transfer from b-AsP/Au side to b-AsP/$MoTe_2$ side, resulting in a negative photocurrent. Note that the near-zero photocurrent in the b-AsP/$MoTe_2$ junction region may be due to the cancellation of the positive and negative photocurrent caused by PTE and PV respectively in the junction. The schematic diagram of the photocurrent generation in the b-AsP/$MoTe_2$ device under local illumination are described in Supplementary Fig. 8e-f.

Based on the above results, the schematic diagram of the photocurrent generation in the b-AsP/$MoTe_2$ device under visible/NIR and MIR global illumination are depicted in Supplementary Fig. 7c and 7d, respectively. Under visible or NIR laser global illumination, both b-AsP and $MoTe_2$ layers generate electron-hole pairs which are separated by the built-in electrical field with direction pointing from b-AsP to $MoTe_2$ side at the junction. The photo-generated electrons and holes move toward b-AsP and $MoTe_2$, respectively, which contributes to the negative photovoltaic photocurrent. Under MIR laser global illumination, an unbalanced lattice temperature distribution is generated in b-AsP layer due to the asymmetric contacts of b-AsP with $MoTe_2$ and Au electrode. The lattice temperature of b-AsP at the $MoTe_2$ conduct side is higher than that at Au electrode contact side because the Seebeck coefficient of b-AsP is higher than that of $MoTe_2$ and the thermal conductivity of $MoTe_2$ is lower than that of Au. Such lattice temperature distribution promotes the diffusion of holes in the b-AsP from the $MoTe_2$ contact side to Au electrode contact side, thus forming a positive PTE photocurrent under zero bias with b-AsP as the source terminal. In addition, we believe that the photocurrent generation mechanism of the as-fabricated b-AsP/$MoTe_2$ devices is not dependent on the thickness and overlap area, because similar photoresponse characteristics are observed in devices with different thicknesses and overlap area (Supplementary Fig. 8, 15, 16).

Supplementary Note 3. Photodetection performance of the b-AsP/$MoTe_2$ heterostructures

Photoresponse rate is a key figure of merit of photodetectors. Supplementary Fig. 13a and 13b presents the photoswitching behavior and response rate of the b-AsP/$MoTe_2$ heterostructure under 730 nm and 4.6 μm illumination at V_{DS} = 0 V, respectively. There is no obvious declination of the photoresponse amplitude as the NIR pulse frequency increases from 2.5 kHz to 25 kHz and MIR pulse frequency increases from 2 to 3 kHz, indicating the potential of high-speed operation. The NIR and MIR photoresponse rate of the heterostructure are as fast as 600 ns/3.7 μs and 2.3 μs /20 μs, respectively, which are faster than most PV and PTE photodetectors.[11-13] In addition, two other important figure of merits, namely responsivity (R) and detectivity (D*), are also calculated by the following equations:[11]

$$R = \frac{I_{ph}}{P \times A} \quad (3)$$

$$D^* = \frac{(A \times B)^{0.5}}{\mathrm{NEP}} \quad (4)$$

Where I_{ph}, P, A, B and NEP refer to photocurrent, incident power density, the effective device area, measuring bandwidth and noise equivalent power, respectively. The effective device areas of the five devices are listed in Supplementary Table 1. Note that the visible/NIR photoresponse is mainly contributed by the b-AsP/$MoTe_2$ junction areas, while the MIR photoresponse mainly come from b-AsP. So, the junction areas and b-AsP areas are used to calculate the visible/NIR and MIR performance, respectively. The NEP can be obtained following NEP = i_N/R, where i_N is noise current density. The noise spectral densities of the as-fabricated five devices are displayed in Supplementary Fig. 14, which are all measured at V_{DS} = 0 V. Based on this, the NEP, R and D* of the five devices under 730 nm and 4.6 μm illumination with various power densities are accordingly calculated, which are shown in Supplementary Fig. 15 and 16, respectively. Supplementary Table 1 present the comparison of photoresponse performance of the five b-AsP/$MoTe_2$ devices with various thicknesses. As can be seen that the responsivity and detectivity show a decreasing trend with thinning thickness, which may be

attributed to the weaker optical absorption of thinner device. For 4.6 μm, a competitive responsivity of 1.13 mA/W with a high detectivity of 9.6×10^8 cm·Hz$^{0.5}$/W is obtained in b-AsP/$MoTe_2$ (device 1) with thickness of 40/50 nm under the power density of 80.21 W/cm^2. In terms of 730 nm, the values of R and D^* are about 0.88 A/W and 4.1×10^{11} cm·Hz$^{0.5}$/W at the power density of 226.64 mW/cm^2, respectively. These figure of merits are competitive in both MIR and NIR photodetectors based on 2D van der Waals heterostructures.[14]

Furthermore, the photoresponse performance of the b-AsP/$MoTe_2$ heterostructure (device 1) under 532 nm and 1470 nm laser illumination are evaluated, as shown in Supplementary Fig. 17. What's more, the device demonstrates broadband MIR photodetection capability up to 10.5 μm at V_{DS} = 0 V, as shown in Supplementary Fig. 18 and 19. The responsivity and detectivity of the heterostructure monotonously decrease from 1.7 to 0.5 mA/W and 1.1×10^9 to 3.1×10^8 cm·Hz$^{0.5}$/W as the wavelength increases from 4.5 to 10.5 μm, respectively, which is attributed the decreasing MIR absorption of b-AsP at the longer wavelength.

In addition, the b-AsP/$MoTe_2$ heterostructure (device 1) demonstrates stable and repeatable photoresponse under simultaneous MIR and NIR illumination, as shown in Supplementary Fig. 20. Note that the simultaneous illumination is carried out by using continuous 4.6 μm laser and pulsed 730 nm laser. It can be observed that the device maintains fast and stable photoresponse under pulsed 730 nm illumination at a frequency of 20 kHz, which is attributed to the fast NIR photoresponse rate. Moreover, the stable photoresponse can be still maintained under 730 nm laser illumination with a frequency of 100 kHz (Supplementary Fig. 21). Such a fast and stable response makes it possible to generate higher spiking rates and provides a guarantee for high-precision MIR intensity coding.

Supplementary Note 4. Rules for determining the encoding parameters for high precise encoding.

Supplementary Fig.24 shows the schematic diagram about how to determine the suitable encoding parameters (mean (u), variance (σ) and spiking threshold current (I_{TC})) to realize high encoding precision. If the linear region of encoding transfer curve is shifted to the middle of the MIR power range of interest, the corresponding parameters can be regarded to be optimal. The rules of setting the encoding parameters are given as follows:

First, we need get the function of photocurrent (I_{DS}) as P_{MIR} and P_{NIR}, of which the schematic is shown in Supplementary Fig. 24a. Then, we need determine the largest P_{MIR} range of interest to encode. We give two examples, a relatively large dynamic range [A, B] and a relatively low dynamic range [A', C]. Their corresponding ranges of I_{DS} when adding NIR light with P_{NIR} ranging from 0 to p_7 are highlighted by red and dark blue dashed parallelogram, respectively. Next, we start to determine u and σ of Gaussian distribution for sampling the NIR light. In real operation, we want to fully exploit the available NIR power range to decrease the demand on the fineness of NIR laser for tuning its optical power. So, u is set to $p_7/2$ to allow the distribution of $I_{DS,B}$ and $I_{DS,A}$ to occupy whole available I_{DS} range, depicted in Supplementary Fig. 24b. The distributions of $I_{DS,B} = N(u_B, \sigma_B)$ and $I_{DS,A} = N(u_A, \sigma_B)$. To make the linear region of encoding transfer curve in the middle of the dynamic range, the value of I_{TC} is set to $(u_B+u_A)/2$, which is roughly estimated as

$$I_{TC}=\left(\frac{I_{DS}(B,p_1)+I_{DS}(B,p_7)}{2}+\frac{I_{DS}(A,p_1)+I_{DS}(A,p_7)}{2}\right)/2 \quad (5)$$

that is convenient for us to directly extract it from the I_{DS} curve. For a relatively low P_{NIR} range, the high precise encoding can be realized by properly decreasing u and σ at a fixed I_{TC}. The final encoding transfer curve is given in Supplementary Fig. 24d-e.

Supplementary Note 5. Analytical results of the device and spiking neural network

Response speed to NIR light in our device is a critical metric that determines how fast our device can encode input MIR signal, and how many time-steps in one trail of NIR optical pulses that the devices can afford within a fixed encoding time. The rising time of our device to NIR light is 600 ns, and the falling time is 3.7 μs. The Fast response speed allows the sampling period (T_S) is reduced to 10 μs, by which the encoding performance has already shown in Fig. 2 of main text. For comparison, the encoding performance of our device at T_S = 100 μs is presented in Supplementary Fig. 25. The time-steps are 10 if the encoding time is fixed to 1 ms. The inadequate time-steps hinders correct representation of MIR power, reflected as irregular nonlinearity in the encoding transfer curve and serious error of spike rate in Supplementary Fig. 25f. We also do the simulation that encodes the intensity of a clock image using time-steps of 10 and 100, respectively. The results indicate that more time-steps thanks to high response speed of the device enable highly-precise encoding.

A current response model of our device to P_{MIR} and P_{NIR} can be fitted by measured data. By this model, simulation results of spike rate as a function of P_{MIR} is given in Supplementary Fig. 26a-c. It's observed that a higher σ can extend to cover P_{MIR} range from 0 to 80.21 W/cm^2. The location of dynamic working range moves with u and I_{TC}.

As shown in Supplementary Fig. 27a-b, more hidden neurons improve the classification ability of SNN traded from the structure complexity. Membrane potential decay rate (β) means the ability of the synaptic neuron memorizing the former information. When β reaches 100%, the accuracy exceeds 95% thanks to ideal memory of neurons, but a real synaptic device hardly has β of 100% without any memorized voltage decay, so the β in this work is set to 95%. The u, σ, I_{TC} and sampling points for two figures are 130 mW/cm^2, 55 mW/cm^2, 0 nA and 50. Supplementary Fig. 27c verifies that training parameters and iteration numbers of SNN is sufficient for loss convergence without underfitting problem. Additionally, the loss convergence of test set proves the SNN has no over-fitting problem.

The impact of other distributions for sampling NIR light on encoding precision

and recognition accuracy of SNN are investigated in Supplementary Fig. 29. To imitate the stochasticity and help device to have a good noise-tolerance, we sample the NIR light with the Gaussian distribution to match the distribution of the noise in opto-electric receivers. Most kinds of noises in opto-electric receivers, such as shot noise and thermal noise, are white noises following the normal Gaussian distribution. That's also why we do not consider other Gaussian distributions like sub-Gaussian and super-Gaussian distributions. In addition, sub-Gaussian and super-Gaussian distributions have an uncertain super-parameter: fourth-order moment, which needs extra discussion that will increase the complexity and uncertainty of our encoding algorithm. Regarding to other distributions, only the distributions with adjustable mean and variance are considered here. The distributions without the two parameters such as Poisson's distribution cannot endow the device with the eye's visual adaptivity to different light intensity. Here, we discuss three different distributions, for instance, Gaussian distribution, uniform distribution, and Laplace distribution. Their sampling sequence, distribution curve and the encoding transfer curve under additive white noise with variance of 10 mW/cm^2 are compared in Supplementary Fig. 29. It's found that all distribution can realize encoding functions, but the Gaussian distribution has the highest noise-tolerance with the smallest error in spike rate under the same additive white noise.

Regarding to the recognition accuracy, the results are show in Supplementary Fig. 29d-l, when the encoding parameters (u, σ, R) are optimized, three distributions have the similar highest recognition accuracy up to 96.7% for high-P_{max} objects. However, the Uniform distribution has the highest recognition accuracy for the objects with P_{max} lower than 20 W/cm^2 followed by Gaussian distribution and Laplace distribution. This is because the SNN is trained by ideal linear encoding. The linearity of encoding transfer curve using Uniform distribution is better than that of other two distributions, and therefore the Uniform distribution matches better to the trained SNN even its encoding precision is lower than Gaussian distribution. Nevertheless, it's noted that the Uniform distribution poses a big challenge on the resolution of output optical power for the NIR laser if time steps increase. Considering the resolution of the output optical power for our laser is only 0.1 mW that limits the use of Uniform distribution in a

relatively low encoding range, we choose Gaussian distribution in the main text to demonstrate our concept and the functionality of our device despite of a little performance sacrifice on the digit recognition task.

The impact of different devices thicknesses and the different wavelengths of stochastic light sources are investigated in Supplementary Fig. 30-31. The results reveal that the devices at different thicknesses and different wavelengths of stochastic light source can possess similar encoding and recognition accuracy if encoding parameters including u, σ and I_{TC} are optimized.

Reference

1 Keum, D. H. *et al.* Bandgap opening in few-layered monoclinic $MoTe_2$. *Nature Phys.* **11**, 482-486, (2015).

2 Liu, B. *et al.* Black arsenic-phosphorus: layered anisotropic infrared semiconductors with highly tunable compositions and properties. *Adv. Mater.* **27**, 4423-4429, (2015).

3 Liu, Y. *et al.* Approaching the Schottky-Mott limit in van der Waals metal-semiconductor junctions. *Nature* **557**, 696-700, (2018).

4 Cheng, R. *et al.* High-performance, multifunctional devices based on asymmetric van der Waals heterostructures. *Nat. Electron.* **1**, 356-361, (2018).

5 Liu, M. *et al.* High yield growth and doping of black phosphorus with tunable electronic properties. *Mater. Today* **36**, 91-101, (2020).

6 Karki, B., Rajapakse, M., Sumanasekera, G. U. & Jasinski, J. B. Structural and thermoelectric properties of black arsenic-phosphorus. *ACS Appl. Energy Mater.* **3**, 8543-8551, (2020).

7 Shafique, A. & Shin, Y. H. Strain engineering of phonon thermal transport properties in monolayer 2H-$MoTe_2$. *Phys. Chem. Chem. Phys.* **19**, 32072-32078, (2017).

8 Zulfiqar, M., Zhao, Y., Li, G., Li, Z. & Ni, J. Intrinsic thermal conductivities of monolayer transition metal dichalcogenides MX_2 (M = Mo, W; X = S, Se, Te). *Sci. Rep.* **9**, 4571, (2019).

9 Dai, M. *et al.* High-performance, polarization-sensitive, long-wave infrared photodetection via photothermoelectric effect with asymmetric van der Waals contacts. *ACS Nano* **16**, 295-305, (2022).

10 Chaves, A. *et al.* Bandgap engineering of two-dimensional semiconductor materials. *npj 2D Mater. Appl.* **4**, 29, (2020).

11 Long, M. *et al.* Room temperature high-detectivity mid-infrared photodetectors based on black arsenic phosphorus. *Sci. Adv.* **3**, e1700589, (2017).

12 Lu, X., Sun, L., Jiang, P. & Bao, X. Progress of photodetectors based on the photothermoelectric effect. *Adv. Mater.* **31**, 1902044, (2019).

13 Wang, F., Pei, K., Li, Y., Li, H. & Zhai, T. 2D homojunctions for electronics and optoelectronics. *Adv. Mater.* **33**, 2005303, (2021).

14 Guan, X. *et al.* Recent progress in short- to long-wave infrared photodetection using 2D materials and heterostructures. *Adv. Optical Mater.* **9**, 2001708, (2020).
